# Accurate intercensal estimates of energy access to track Sustainable Development Goal 7

**DOI:** 10.1140/epjds/s13688-022-00371-5

**Published:** 2022-12-09

**Authors:** Neeti Pokhriyal, Emmanuel Letouzé, Soroush Vosoughi

**Affiliations:** 1 Dartmouth College, Hanover, USA; 2 Data-Pop Alliance, New York, USA; 3 University Pompeu Fabra, Barcelona, Spain

**Keywords:** Clean energy access, Gaussian processes, Earth-observation data, Sustainable Development Goals

## Abstract

Intercensal estimates of access to electricity and clean cooking fuels at policy planning microregions in a country are essential for understanding their evolution and tracking progress towards Sustainable Development Goals (SDG) 7. Surveys are prohibitively expensive to get such intercensal microestimates. Existing works, mainly, focus on electrification rates, make predictions at the coarse spatial granularity, and generalize poorly to intercensal periods. Limited works focus on estimating clean cooking fuel access, which is one of the crucial indicators for measuring progress towards SDG 7. We propose a novel spatio-temporal multi-target Bayesian regression model that provides accurate intercensal microestimates for household electrification and clean cooking fuel access by combining multiple types of earth-observation data, census, and surveys. Our model’s estimates are produced for Senegal for 2020 at policy planning microregions, and they explain 77% and 86% of variation in regional aggregates for electrification and clean fuels, respectively, when validated against the most recent survey. The diagnostic nature of our microestimates reveals a slow evolution and significant lack of clean cooking fuel access in both urban and rural areas in Senegal. It underscores the challenge of expanding energy access even in urban areas owing to their rapid population growth. Owing to the timeliness and accuracy of our microestimates, they can help plan interventions by local governments or track the attainment of SDGs when no ground-truth data are available.

**Supplementary Information:**

The online version contains supplementary material available at 10.1140/epjds/s13688-022-00371-5.

## Introduction

Access to energy directly translates into a multitude of factors affecting human development that includes education, health, gender equality, clear air and water [1]. Yet, globally 840 million people live without electricity and 3 billion people cook using traditional fuels [2]. Even before the COVID-19 crisis, it was projected that around 620 million people would still lack access to electricity in 2030 with 85% percent of them in Sub-Saharan Africa and 2.3 billion people would still not have access to clean cooking fuel [3]. The COVID-19 pandemic threatens the progress that has been made towards the United Nations Sustainable Development Goal (SDG) 7 on affordable and clean energy access [4]. *Access to electrification and clean cooking fuels* are the two main indicators instrumental for measuring progress towards SDG 7, and is the focus of this work. Accurate tracking of SDG 7 is dependent on frequent and detailed micro-regional data, with special focus on clean cooking fuel access [5, 6].

Since census and surveys are labor-intensive, cost millions of dollars and involve a lag of multiple years to get updated results, researchers are studying earth observation (EO) data owing to its high revisit rate to understand various facets of energy accessibility, grid structure, supply and demand [7–11]. Most extant works on household energy access have studied the status of electrification and are limited to coarse spatial granularity of countries or for sparse villages at continent scale for a single time-point [12–14]. These estimates are validated for time-points coinciding with surveys, when training and validation data are readily available. There seems to be scant work in studying the temporal evolution of these estimates beyond the survey years, paradoxically when these are most needed, except a study by [7] which reports poor results for electrification access. Therefore, there is an imminent need for methods that can accurately measure, track and nowcast population wide energy access during intercensal periods in a cost-effective manner. *Nowcasting* is defined as the process of getting intercensal estimates of energy access. Importantly, extant works on energy access focus on binary variables related to electrification. There seems to be scant works in understanding the cooking fuel accessibility at microregional scale, as current studies [15, 16] focus on global and country-wide access of clean cooking fuels.

We propose a novel *spatio-temporal multi-target Bayesian regression framework* that reliably nowcasts household energy access for both the lighting and cooking needs, at microregions using multiple types of publicly available EO datasets, namely nighttime lights, aerosol optical depth data and Landsat-8 satellite imagery, and census and surveys. We focus on indicators critical for tracking SDG 7 – *access to electrification and clean cooking fuel for a household*. Our model learns the complex relationship between features derived from EO and energy access targets for the censal year and, also, leverages data from georeferenced surveys conducted in subsequent years, to provide reliable nowcasts for intercensal periods.

We observe a positive correlation between a household’s access to electricity for lighting and liquefied petroleum gas (LPG) for cooking, and a negative correlation between its electrification and use of lamp for lighting or wood for cooking (see Additional file 1 Figure 1b). We exploit these correlations among the energy access indicators by formulating our problem as a multi-target regression, where the goal is to simultaneously learn multiple targets given a single input observation [17, 18]. Learning multiple targets (outputs) is shown to be beneficial when the outputs are multi-variate and when complex inter-dependencies exist among them. In these scenarios, multi-target regression is shown to provide better predictive performance, robustness to noise and missing data, and computational efficiency [19].

To facilitate insights into *inequities*, we model the delineation of energy access along the urban-rural divide in our Bayesian framework. As a measure of our model’s generalization in time, we *validate* our intercensal microestimates using the temporally closest DHS surveys.

Our model’s microestimates are produced at policy planning microregions, called *communes* in Senegal, for intercensal years, *2015, 2017 and 2020*, and are validated using concurrent DHS data.^1^ On average, our nowcasts can explain >77% and >71% of variation in regional aggregates for electricity and clean cooking fuel access, respectively. For 2020, we report a Pearson’s r correlation of 0.88 for electricity and 0.92 for clean cooking fuel access between our estimates and DHS data. Our results expose stark disparities in energy attainment for communes delineated along the urban-rural divide as well as juxtaposed against their population growth. Our model *simultaneously* quantifies the evolution of all household energy access indicators in Senegal, e.g., wood, coal and kerosene lamps, thus providing the policy makers with a complete spectrum of energy accessibility.

In summary, our *contributions* are as follows:

1. We propose a novel *spatio-temporal multi-target Bayesian regression* model that accurately estimates the entire spectrum of household energy access at microregions using multiple types of publicly available EO datasets in Senegal for the intercensal periods. Two important distinctions of our model compared to existing works are as follows: Understanding a *multi-spectrum access* for household energy (compared to a mostly binary notion of presence/absence of electricity) by proposing a multi-target regression model and second, the use of *aerosol data* for energy access has not been explored yet.
2. We validate the reliability of the microestimates of our model for several intercensal years and report highly accurate results at regional levels both for spatial cross-validation and for intercensal years. For 2020, our model’s errors are consistently better than the existing best-performing model’s estimates for electricity and clean cooking fuel access.
3. We demonstrate the significant *disparities* in energy access for urban and rural areas, and juxtapose them against the population growth and provide insights for policy makers into the evolution and the challenges in household energy access in Senegal. Such insights are possible because we built a *specialized kernel* that explicitly models the urban-rural delineation along with spatial and temporal effects.
4. We model the accessibility of *cooking fuel*, which is a critical indicator for SDG 7.1 using multiple disparate data sources, and there seems to be scant work in understanding its access. Lack of clean fuel for cooking disproportionally impacts women and children, their educational attainment and their indoor air pollution, and the problem is exacerbated for poorer and vulnerable communities.

### A note on definition of energy accessibility

Most existing studies have focused on a binary definition of energy access, i.e., if a household (or village) has access to electricity or not, by measuring binary responses to questions like, “does the household have electricity connection?” or “cooking with non-solid fuels?”. However, this approach fails to capture the full spectrum of lighting and cooking fuel access for a household and recent works calls to move beyond such mono-dimensionality [7]. Hence, we adopt a *multi-dimensional* view of energy access at micro-regional level as determined by the census of that country, thus providing policy makers with more nuanced information about diverse sources of energy employed by the population for lighting and cooking in their homes. For Senegal, the prominent modes of lighting are candle, electricity, lamp and for cooking are coal, gas, wood. Therefore, rather than a single value, the energy access for a microregion is defined to be a vector whose length corresponds to the number of prominent categories of lighting and cooking modes, and each entry contains the fraction of households (in that microregion) that use that particular category of lighting and cooking.

The rest of the paper is organized as follows: Sect. 2 describes existing works that deal with estimated energy access using multiple data sets. Section 3 describes details of the data used in this study and Sect. 4 details the model and the inference procedure. Section 5 describes the validation and results for the target country of Senegal. Section 6 provides further discussions, including the limitations of this study and future directions.

## Related works

Extant works on household energy access are, mostly, limited to studying binary notion of electrification [12–14, 20] using predominantly nighttime light data and producing estimates at a given time point. Most of these works provide promising estimates at time-points coinciding with surveys, but it is unclear how they will generalize to intercensal time periods. There are very few studies to determine if the inferential relationships learnt will be robust over time – a need that has been highlighted by recent surveys on using satellite imagery for sustainable development [11, 21]. A recent work [7] maps the spatial heterogeneity of national electricity access from 2014-2019 for the Africa, but yields poor temporal generalization. While there are longitudinal studies mapping the evolution of electrification over time, but these are retrospective in nature, rather than a nowcasting model [12, 22, 23].

The existing models mostly study the binary access to electrification as this metric is easily interpretable. However, owing to issues of reliability of connection and affordability, binary metrics may obfuscate the nuanced ways in which households have access to energy [7].

The existing works to understand energy access from EO data mostly employ generalized linear models as these models provide interpretability [7, 22, 24]. Some top performing machine learning methods for electrification prediction task are gradient boosting classifiers [25, 26], logistic regression [27], Gaussian Process (GP) classification [12, 27]. We *compare* our proposed model with each of these existing works.

Besides electrification, researchers have explored different satellite data products, like Landsat-8, Sentinel data, population data, for predicting developmental indicators, such as roof types [26], drinking water [14], poverty mapping [28–30] etc. Recent works [12, 14] have employed such data sets for electricity infrastructure prediction and household energy electrification prediction (again a binary notion) at continent wide scale and provide promising results using deep learning based approaches, based on a convolutional neural network(CNN). These approaches are not directly applicable for our problem setting, as they require substantial amounts of training data and we deal with only a handful of micro-regions for a given country, instead of thousands of villages spread across the entire continent of Africa.

Owing to the better predictive power of CNN-based features extracted from satellite images over simpler features, we employ the state-of-art deep learning model based on the ResNet-18 architecture [31], as our choice for feature extraction from satellite imagery. While researchers have pointed to the tradeoff between performance and interpretability with deep learning models, by using them as feature extractors in our Bayesian model, we weave interpretability into our modeling framework and provide *insights* useful to policy planners.

Additionally, most of these works provide estimates at coarse spatial granularity e.g. for villages spread across the entire African continent [31]. However for SDG monitoring countries need such estimates at policy-planning level as explored in this work [1]. A critical challenge, here, remains the unavailability of disaggregated statistical data from census and surveys.

## Data

In this section, we describe the EO data sets used in this study, starting with a description of the target country. We then outline the procedure of extracting the various covariates from the EO data sets and calculating the energy access targets from census and survey data sets.

### Country details

The study is conducted for *Senegal*, a Sub-Saharan country which is categorized with low human development. According to the 2022 Tracking SDG7 report, Senegal has an electrification access rate of 71% and national access to clean cooking solutions at 31%. It ranks 170 out of 191 countries on the Human Development Index in 2021 [32]. Lack of electricity supply is one of the main constraints hindering Senegal’s socio-economic development. The remote and rural areas lack access to modern energy services, face frequent power cuts that lower the quality of life of the poor and vulnerable communities and reduce business efficiency [33]. Regarding cooking fuel access, rural areas are highly dependent on wood, while urban populations mostly use coal and, less frequently, gas.

### Data

The following data sets were used for this study. See Table 1 for details regarding data procurement, and the details for feature extraction are given below:

1. *Census data*: We use a 10% sample of the most recent census (called RGPHAE (*Recensement General de la Population de l’Habitat de l’Agriculture et de l’Elevage*)), provided by *Agence Nationale de la Statistique et de la Demographie* (ANSD), which is the National Statistics Office of Senegal. It was conducted in 2013 and was made available in 2015. The data is evenly sampled across the entire population of Senegal, with data from 1.4 million individuals, spread across 150,000 households. It represents the most spatially detailed and comprehensive coverage of national statistics and has information about household features including mode of lighting and type of cooking fuel.
2. *Demographic and Household survey data* (DHS): These surveys collect a multitude of information across varied topics of interest for a population sample that participates in the DHS program. These are based on sampling clusters, which collect information for individuals or household records. For privacy reasons, cluster locations are displaced up to 2 km for urban areas and up to 5 km for rural areas, about 1% of which can be displaced up to 10 km [34]. The cluster locations for DHS corresponding to 2015, 2017 and 2019 are shown in Additional file 1 Fig. 1.
3. *Nighttime lights* (NTL) capture the radiance associated with lights at night and is often used in studying electrification access at various spatial heterogeneity [10, 35–37]. We use an integrated publicly available NTL dataset across the years [38].
4. *Aerosol Optical Depth* (AOD) is extracted from the Moderate Resolution Imaging Spectroradiometer (MODIS) on NASA’s Terra and Aqua satellites, and captures the aerosol content over a spatial location. A median composite of the annual AOD data is taken to mitigate the effect of seasonal dust storms.
5. *Landsat-8 satellite data*: This data has been shown to predict infrastructural qualities, especially those related to electrification [14] in Africa, when compared to nightlights and Sentinel 1 satellite data. To extract features from this data, we employed a pretrained deep neural network, based on ResNet-18 architecture and adapted for multispectral satellite imagery. This model has been shown to outperform other models in extracting features to predict asset wealth (that includes household indicators including electrification and possession of assets like television, phone, etc.) [31]. We use the intermediate activations from the penultimate layer in the deep neural network as features that, likely, correspond to information related to urban infrastructure, agricultural land and other land forms (like desert and water bodies). We use 1-year median composite images for Senegal. The input to the deep neural network is the 7 band image of size $(224 \times 224)$ and the output is a 512 length vector corresponding to the activations from the penultimate layer. The composite Landsat-8 image, in which each pixel corresponds to a 30 sq. m. area on ground, is divided into “tiles” of size $(224 \times 224)$, each corresponding to an area of 6.72 sq. km. Each tile is fed into the deep neural network and transformed into a 512 length feature vector.

**Table 1:** Description of disparate data sets used in the study. The first two rows corresponding to census and survey data are used to create targets for our model. The next three rows, NTL, AOD and L8 are EO datasets used to extract covariates. The last row corresponding to population data is used in getting settlement information and is used in weighting the covariates of EO features. DMSP/OLS/VIIRS refers to the Defense Meteorological Satellite Program/Operational Linescan System (OLS) and the Visible Infrared Imaging Radiometer Suite (VIIRS)

Datasets & resolution	Frequency of availability	Source	Raw covariates per pixel per year	Details	Cost for data collection & preparation
Targets					
Senegal Census	Every 10 years	National Statistics Office of Senegal (ANSD)	NA	Energy access indicators	$$$ (USD 29 million)
DHS Survey Data	2-5 years	United States Agency for International Development (USAID)	NA	Energy access indicators	$$ (but publicly available)
EO Data					
Harmonized Night-time Lights (*NTL*) (1 km/pixel resolution)	Monthly	Harmonized Nightlight Dataset [38]	Mean NTL averaged annually	NTL data DMSP/OLS/VIIRS	Low/no cost (data exhaust)
Aerosol Optical Depth (*AOD*) (1 km/pixel resolution)	Monthly	Google Earth Engine	Median AOD averaged annually	Blue band (0.47 *μm*) AOD over land	Low/no cost(data exhaust)
Landsat-8 multi-spectral imagery (*L8*) (30 m/pixel resolution)	Monthly	Google Earth Engine	512 length vector extracted using a deep neural network [31]	Bands – Red, Green, Blue, Near Infrared (NIR), Shortwave Infrared 1 & 2 (SWIR1/2), Thermal (TEMP1)	Low/no cost (data exhaust)
Human settlement					
Population estimates (100 m/pixel resolution)	Yearly	WorldPop [39]	NA	High resolution gridded population	$$ (but publicly available)

### Creating microregional covariates from EO data

The raw covariates for each of NTL, AOD and L8 data sets listed in Table 1 are aggregated to microregions, using a *population weighted aggregation scheme* to capture the per-household behavior in that microregion, which is empirically shown to provide better performance in estimating household energy access compared to extant works. Our scheme is outlined here. Note that for each EO data set, the geographical area corresponding to each pixel is different.

The covariates from EO data are extracted at the granularity of pixels, while our analysis is performed at policy planning microregions. Spatially, a microregion is composed of a number of pixels. While some pixels lie entirely within the spatial extents of a microregion, others may fall at its boundary with neighboring microregions. We follow a specific aggregation scheme to get the EO covariates for a microregion, outlined below.

For each EO covariate (*f*), we calculate the *weighted mean* $\mu _{fc}$ and *weighted variance* $\sigma ^{2}_{fc}$ for a given microregion, *c*, as follows:

1 $$\begin{aligned}& \mu _{fc} = \frac{\sum_{\forall i}{f_{i} \times \bar{p}_{ic}}}{\sum_{\forall i}{\bar{p}_{ic}}}, \end{aligned}$$

2 $$\begin{aligned}& \sigma ^{2}_{fc} = \frac{\sum_{\forall i}{(f_{i} - \mu _{fc})^{2} \times \bar{p}_{ic}}}{ \sum_{\forall i}{\bar{p}_{ic}}}, \end{aligned}$$

where $f_{i}$ corresponds to the covariates for a pixel (indexed by *i*). $\bar{p}_{ic}$ is an area-adjusted population of the pixel, calculated as $\bar{p}_{ic} = p_{i}\frac{a_{ic}}{a_{i}}$, where $p_{i}$ is the population for the pixel, $a_{i}$ is the geographical area of the pixel and $a_{ic}$ is the geographical area of the pixel contained within the microregion *c*. Note that $\bar{p}_{ic}$ is 0 for pixels that do not have any overlap with microregion *c*. The population count, $p_{i}$, is obtained by resampling the gridded population data to the appropriate spatial resolution for the feature *f*.

While the numerator in (1) weights the EO covariates for pixels by underlying population, dividing it by the total population of the microregion, ensures that the features capture the per-household behavior for that microregion. We also estimate the variance corresponding to each aggregated EO covariates for a microregion as given in (2). It captures the noise that is attributed when EO covariates are aggregated to microregions.

Finally, there are 2 covariates corresponding to *mean and variance* of NTL and 2 for AOD. For the Landsat-8 high dimensional feature vector, we use Principal Component Analysis (PCA) to reduce them to 20 covariates by mapping the data to the top 20 principal components that retains 95% of the data variance. *Dimensionality reduction* is often done for computational efficiency and to prevent overfitting in small datasets. The corresponding variances associated with each of these features is mapped in the same manner, giving 40 covariates (mean and variances) from L8 imagery.

### Creating energy access targets from census and DHS data

#### Energy access targets from census

In Senegal’s 2013 census, the major categories for lighting, in order of popularity, are *electricity*, *rechargeable lamp*, *candles* and others; while those for cooking fuel are *wood*, *coal*, *gas* and others. Each household identifies as using a specific category of lighting and cooking fuel. We create an 8 length accessibility vector for each microregion corresponding to 4 categories each of lighting and cooking fuel. The exact mapping of each census response to this vector is detailed in Additional file 1 Section “Mapping of census responses”. Each entry in the vector contains the fraction of households using that particular category of lighting/cooking fuel within the microregion. The household responses are weighted by their sampling coefficients provided in the census to make them representative of the population.

#### Energy targets from DHS

DHS data occur for select clusters throughout the country, whose locations change for every new survey. For each DHS survey, the geocoded clusters are assigned to their spatially nearest microregion and a 8 length accessibility vector is created by consolidating the household responses related to lighting and cooking fuel access for all clusters that fall within that microregion, using the similar approach described for census above. We weight these responses using the provided sampling weights to account for the selection biases.

## Model description

This section describes the proposed Bayesian model, and details on model training and inference. Since Gaussian Processes (GP) form the basis of our model, a brief background is provided.

### Model intuition

We propose a semi-parametric model given as: ${\textbf{y}} = {\mathbf{B}}{\textbf{x}} + f(\textbf{x},{\mathbf{s}},u, t) + \epsilon $. The first term models the linear relationship between EO covariates (**x**) and the targets (**y**), where **B** is the coefficient matrix for the linear model. The multiple targets of regression correspond to household energy access indicators (e.g., electricity, gas etc.) The second term employs a non-linear functional mapping based on GP between an augmented covariate vector and **y**. The augmented covariate vector includes **x**, the spatio-temporal coordinates (**s**, *t*), and an urban-rural indicator (*u*).

GPs belong to the class of *Bayesian models*, where the choice of kernel functions enables one to learn highly nonlinear relationships between the covariates and target variables [40]. GPs can be made more flexible and interpretable by combining (adding or multiplying or convolving) different kernels, where each kernel models a certain effect within individual covariates.

We propose a specialized kernel for our GP model, with the following form:

3 $$ {\mathbf{K}}_{mo} = \underbrace{{\mathbf{K}}_{c} + ({ \mathbf{K}}_{sp} * {{\mathbf{K}}}_{ur}) + { \mathbf{K}}_{t}}_{ \text{covariate effect}} \odot \underbrace{{ \mathbf{K}}_{\ell}}_{ \substack{\text{multi-target}\\ \text{effect}}}. $$

The first kernel in (3) models three types of effects in an additive form: a *EO covariate effect* ${\mathbf{K}}_{c}$, a spatial auto-correlation effect with urban-rural delineation ${\mathbf{K}}_{sp} * {{\mathbf{K}}}_{ur}$ which assigns more weight to *spatially proximal* and similar microregions (i.e. in EO data, an urban location might derive some similarity from nearby rural locations and also from nearby other urban locations), and a *temporal recency* effect which assigns more weight to recent observations ${\mathbf{K}}_{t}$. The second kernel ${\mathbf{K}}_{\ell}$ provides the *multi-target formalism* by exploiting correlations across different targets.

The rationale for using such specialized kernel is that additive kernels are known to extrapolate well to unseen test data [41, 42], and we empirically demonstrate better performance of our model compared to existing works.

Model training involves estimating the optimal values for the coefficient matrix, **B**, and the hyper-parameters associated with the kernel ${\mathbf{K}}_{mo}$ in (3), and is done by maximizing the marginalized log-likelihood of the training data. Elastic-net regularization is employed on the linear model to prevent learning from spurious features and to avoid overfitting on limited training data [43]. We perform out of sample spatial and temporal validation to test our model’s generalizability.

### Model details

#### Notation

For a given microregion, indexed by *c*, the covariate vector, target vector, spatial coordinates, and the urban-rural indicator, are denoted by ${\mathbf{x}}_{t}^{(c)}$, ${\mathbf{y}}_{t}^{(c)}$, ${\mathbf{s}}^{(c)}$, and $u^{(c)}$, respectively, and are collectively denoted as ${\mathbf{z}}^{(c)}_{t}$. Note that the covariate vectors and target vectors are also indexed by time *t*, denoting the corresponding years. Each individual target will be denoted by $y^{(c)}_{to}$. For notational simplicity, we will drop the superscript *c* to denote a typical microregion, unless needed. In general, we will use a lower-case bold symbol to denote a vector, upper-case bold symbol to denote a matrix, and a lower-case normal symbol to denote a scalar value. Collections (or sets) of entities will denoted using calligraphic symbols, e.g., $\mathcal{X}$, $\mathcal{Y}$. The *o*th entry of a vector, e.g., **x**, will be denoted as $x_{o}$.

#### Model description

The proposed semi-parametric model is written as:

4 $$ {\mathbf{y}}_{t} = {\mathbf{B}} {\mathbf{x}}_{t} + f({ \mathbf{x}}_{t},{\mathbf{s}},u, t) + \epsilon, $$

where **B** is the coefficient matrix for the linear component and *ϵ* denotes the unexplained noise and is modeled as a zero-mean Gaussian random variable, i.e., $\epsilon \sim N(0,\sigma ^{2}_{n})$. The function $f()$ captures the non-linear dependencies between the covariates and the residual vector, $\boldsymbol{\delta}_{t}$, where $\boldsymbol{\delta}_{t} = ({\mathbf{y}}_{t} - {\mathbf{B}}{\mathbf{x}}_{t})$, and is modeled using a Gaussian Process.

##### Background on Gaussian Processes (GP)

GP is a Bayesian formulation to learn non-parametric, non-linear functions, through the use of kernels. A GP allows placing a stochastic prior on the function $f({\mathbf{z}}_{t})$, where ${\mathbf{z}}_{t} \equiv ({\mathbf{x}}_{t},{\mathbf{s}},u, t)$. The GP prior is completely specified by a mean function, $m(\cdot )$, and a positive-definite kernel function $k(\cdot ,\cdot )$. The mean function represents the expected value of $f()$, i.e., $m({\mathbf{z}}_{t}) = \mathbb{E}[f({\mathbf{z}})]$, and is often set to 0, i.e., $m({\mathbf{z}}_{t}) = 0$. The kernel function defines the covariance between any two realizations of $f()$, i.e.,

5 $$ k\bigl({\mathbf{z}}_{t},{\mathbf{z}}^{\prime}_{t} \bigr) = \mathbb{E}\bigl[f({\mathbf{z}}_{t})f\bigl({ \mathbf{z}}^{\prime}_{t}\bigr)\bigr] $$

assuming a zero mean function.

The definition of GP specifies that for any finite collection of inputs, $\mathcal{Z} = ({\mathbf{z}}^{c_{1}}_{t_{1}},{\mathbf{z}}^{c_{2}}_{t_{2}}, \ldots ,{\mathbf{z}}^{c_{n}}_{t_{n}})$ the vector of function values, ${\mathbf{f}}(\mathcal{Z}) = (f({\mathbf{z}}^{c_{1}}_{t_{1}}),f({\mathbf{z}}^{c_{2}}_{t_{2}}), \ldots , f({\mathbf{z}}^{c_{n}}_{t_{n}}))$, follow a multivariate Gaussian distribution, i.e.,

6 $$ {\mathbf{f}}(\mathcal{Z}) \sim N({\mathbf{0}},{\mathbf{K}}_{\mathcal{Z},\mathcal{Z}}), $$

where ${\mathbf{K}}_{\mathcal{Z},\mathcal{Z}}$ is a $(n \times n)$ covariance matrix, such that the *ij*th entry is equal to $k({\mathbf{z}}^{c_{i}}_{t_{i}},{\mathbf{z}}^{c_{j}}_{t_{j}})$.

For a single output, indexed by *o*, a GP regression model (GPR) can be defined by assuming that the targets are modeled as:

7 $$ \boldsymbol{\delta}_{o} \sim N\bigl({\mathbf{f}}(\mathcal{Z}),\sigma ^{2}_{n}{ \mathbf{I}}\bigr), $$

where **I** is the $(n \times n)$ identity matrix. Using (6) and (7), one can marginalize out ${\mathbf{f}}(\mathcal{Z})$, such that:

8 $$\begin{aligned} \begin{aligned} p(\boldsymbol{\delta}_{o}\vert \mathcal{Z}) & = \int p\bigl( \boldsymbol{\delta}_{o}\vert {\mathbf{f}}( \mathcal{Z})\bigr)p\bigl({\mathbf{f}}( \mathcal{Z})\bigr)\,d{\mathbf{f}} \\ & = N\bigl(0,{\mathbf{K}}_{\mathcal{Z},\mathcal{Z}} + \sigma ^{2}_{n}{ \mathbf{I}}\bigr). \end{aligned} \end{aligned}$$

#### Choice of kernel function

Our kernel function is formulated as follows:

9 $$ k\bigl({\mathbf{z}}^{c_{i}}_{t_{i}},{\mathbf{z}}^{c_{j}}_{t_{j}} \bigr) = k_{f}\bigl({\mathbf{x}}^{c_{i}}_{t_{i}},{ \mathbf{x}}^{c_{j}}_{t_{j}}\bigr) + \bigl(k_{sp}\bigl({ \mathbf{s}}^{c_{i}},{\mathbf{s}}^{c_{j}}\bigr) \times k_{ur} \bigl(u^{c_{i}},u^{c_{j}}\bigr)\bigr) + k_{t}(t_{i},t_{j}), $$

where $k_{f}$, $k_{sp}$, $k_{ur}$ and $k_{t}$ denote the kernels that capture the similarity in covariates, spatial autocorrelation, urban-rural delineation and temporal recency. We use *squared exponential* kernel function for $k_{f}$, $k_{sp}$ and $k_{t}$, which is the most widely used kernel function because of its ability to learn smooth non-linear functional relationships [40]. The individual kernel specifications are given as follows:

10 $$\begin{aligned}& k_{f}\bigl({\mathbf{x}}^{c_{i}}_{t_{i}},{ \mathbf{x}}^{c_{j}}_{t_{j}}\bigr) = \sigma _{f}^{2} \exp \biggl({- \frac{ \Vert {\mathbf{x}}^{c_{i}}_{t_{i}}-{\mathbf{x}}^{c_{j}}_{t_{j}} \Vert ^{2}}{2\ell _{f}^{2}}} \biggr), \end{aligned}$$

11 $$\begin{aligned}& k_{sp}\bigl({\mathbf{s}}^{c_{i}},{\mathbf{s}}^{c_{j}} \bigr) = \sigma _{sp}^{2} \exp \biggl({- \frac{ \Vert {\mathbf{s}}^{c_{i}}-{\mathbf{s}}^{c_{j}} \Vert ^{2}}{2\ell _{sp}^{2}}} \biggr), \end{aligned}$$

12 $$\begin{aligned}& k_{t}(t_{i},t_{j}) = \sigma _{t}^{2} \exp \biggl({- \frac{(t_{i}-t_{j})^{2}}{2\ell _{t}^{2}}} \biggr). \end{aligned}$$

The urban-rural delineation is modeled by $k_{ur}$, which is specified as the following categorical kernel:,

13 $$ k_{ur}\bigl(u^{c_{i}},u^{c_{j}}\bigr) = \textstyle\begin{cases} 1 & \text{if } u^{c_{i}} = u^{c_{j}}, \\ 0 & \text{otherwise}. \end{cases} $$

The scalars $\sigma _{f}$, $\ell _{f}$, $\sigma _{sp}$, $\ell _{sp}$, $\sigma _{t}$, $\ell _{t}$ are the hyper-parameters of the kernel functions and are estimated from the data, as described later.

##### Feature selection

To perform feature selection on EO data, we employ Automatic Relevance Determination kernel (ARD) on our model. ARD kernels are effective in selecting a smaller explanatory subset of features from a large set of irrelevant features by regularizing the solution space using a data-dependent prior [44]. Note that the feature kernel in (10) uses a single global characteristic length scale ($\ell _{f}$). However, for ARD each feature has a different characteristic length scale, denoted by $\ell _{fr}$ for the *r*th feature. The feature kernel for ARD is given as:

14 $$ k_{f}\bigl({\mathbf{x}}^{c_{i}}_{t_{i}},{ \mathbf{x}}^{c_{j}}_{t_{j}}\bigr) = \sigma _{f}^{2} \exp \biggl({-\frac{1}{2}\bigl({\mathbf{x}}^{c_{i}}_{t_{i}}-{ \mathbf{x}}^{c_{j}}_{t_{j}}}\bigr)^{ \top }P^{-1} \bigl({\mathbf{x}}^{c_{i}}_{t_{i}}-{\mathbf{x}}^{c_{j}}_{t_{j}} \bigr) \biggr),\qquad P = \operatorname{diag}(\ell _{f1},\ell _{f2},\ldots ). $$

The inverse of the length scales of each feature, i.e., $\frac{1}{\ell _{fr}}$, is used a proxy for feature relevance [40].

#### Handling multiple targets

The GP regression model described above can only handle a single target. Since the problem studied in this paper involves multiple targets, we present the following scheme, adopted from [45], to exploit the correlations among the targets in the regression model. In this formulation, each instance consisting of a covariate vector and *m* length target vector is converted into *m* instances with a scalar target value. We introduce an additional discrete covariate, *ℓ*, which corresponds to the index of the target. For example, a covariate and a *m* length target vector pair given as $\langle{\mathbf{z}}^{(c)}_{t}; \boldsymbol{\delta}^{(c)}_{t}\rangle $ is transformed into *m* pairs as follows:

15 $$ \bigl\langle {\mathbf{z}}^{(c)}_{t}; \boldsymbol{\delta}^{(c)}_{t}\bigr\rangle \Rightarrow \textstyle\begin{cases} \langle ({\mathbf{z}}^{(c)}_{t}, 1); \delta ^{(c)}_{t1}\rangle, \\ \langle ({\mathbf{z}}^{(c)}_{t}, 2); \delta ^{(c)}_{t2}\rangle, \\ \vdots \\ \langle ({\mathbf{z}}^{(c)}_{t}, m); \delta ^{(c)}_{tm}\rangle. \end{cases} $$

Note that the target is transformed into a scalar. We denote the augmented covariate vector as $\bar{\mathbf{z}}^{(c)}_{to} \equiv ({\mathbf{z}}^{(c)}_{t}, o)$. The extra covariate is handled by multiplying the kernel function, $k()$, in (9) with a target-specific kernel function, $k_{\ell}()$, to obtain the final kernel function:

16 $$ \bar{k}\bigl(\bar{\mathbf{z}}^{c_{i}}_{t_{i}},\bar{ \mathbf{z}}^{c_{j}}_{t_{j}}\bigr) = k\bigl({ \mathbf{z}}^{c_{i}}_{t_{i}},{ \mathbf{z}}^{c_{j}}_{t_{j}}\bigr) \times k_{\ell}(o_{i},o_{j}). $$

Note that the resulting covariance matrix for an augmented single-target data set can be expressed as:

17 $$ {\mathbf{K}}_{\bar{\mathcal{Z}},\bar{\mathcal{Z}}} = {\mathbf{K}}_{\mathcal{Z}, \mathcal{Z}} \otimes { \mathbf{K}}_{\ell }, $$

where ⊗ denotes the Kronecker product between the $(n \times n)$ covariance matrix, ${\mathbf{K}}_{\mathcal{Z},\mathcal{Z}}$ and the $(m \times m)$ matrix ${\mathbf{K}}_{\ell}$, such that $k_{\ell}(o_{i},o_{j}) = {\mathbf{K}}_{\ell}[o_{i},o_{j}]$. For GP, ${\mathbf{K}}_{\bar{\mathcal{Z}},\bar{\mathcal{Z}}}$ needs to be a positive-definite, which means that ${\mathbf{K}}_{\ell}$ should also be positive-definite.

The $m^{2}$ entries in ${\mathbf{K}}_{\ell}$ can be thought of as the hyper-parameters of the kernel function in (16) and can be learnt from the training data. However, instead of treating each entry as a hyper-parameter, we consider a parameterization of ${\mathbf{K}}_{\ell}$ using fewer hyper-parameters. In particular, we consider a *spherical parameterization* [46] of ${\mathbf{K}}_{\ell}$, given as follows:

18 $$\begin{aligned} {\mathbf{K}}_{\ell} = {\mathbf{S}}^{\top }{\mathbf{S}}, \end{aligned}$$

where **S** is an upper triangular matrix of size $(m \times m)$, whose *o*th column contains the spherical coordinates in $\mathbb{R}^{o}$ of a point on the hypersphere, $\mathbb{R}^{(o - 1)}$, followed by $(m-o)$ zeros. For example, for $m = 4$:

19 $$S=\left[\begin{array}{cccc} 1 & \cos\phi^{\left(1\right)} & \cos\phi^{\left(2\right)} & \cos\phi^{\left(4\right)}\\ 0 & \sin\phi^{\left(1\right)} & \sin\phi^{\left(2\right)}\cos\phi^{\left(3\right)} & \sin\phi^{\left(4\right)}\cos\phi^{\left(5\right)}\\ 0 & 0 & \sin\phi^{\left(2\right)}\sin\phi^{\left(3\right)} & \sin\phi^{\left(4\right)}\sin\phi^{\left(5\right)}\cos\phi^{\left(6\right)}\\ 0 & 0 & 0 & \sin\phi^{\left(4\right)}\sin\phi^{\left(5\right)}\sin\phi^{\left(6\right)} \end{array}\right].$$

Here, $\phi ^{(1)}, \phi ^{(1)}, \ldots $ are the hyper-parameters that parameterize the matrix **S**. For *m* targets, one would require $\frac{m(m - 1)}{2}$ hyper-parameters to specify **S**. The spherical parameterization has three advantages. First, it allows us to parameterize a $(m \times m)$ matrix using only $\frac{m(m - 1)}{2}$ hyper-parameters. Second, it ensures that the resulting matrix ${\mathbf{K}}_{\ell}$ is positive-definite. And finally, the off-diagonal entries of ${\mathbf{K}}_{\ell}$ encode the correlation among the targets and can be interpreted as such after training the model.

#### Model training

The parameters of the proposed model consist of the coefficient matrix for the linear model, **B**, the variance term for the observational likelihood in (7), $\sigma _{n}$, the kernel hyperparameters, $\ell _{f}$, $\sigma _{f}$, $\ell _{sp}$, $\sigma _{sp}$, $\ell _{t}$, $\sigma _{t}$ (see (10), (11), (12)), and the spherical coordinates in the upper-triangular entries of **S**.

We assume that the training data consists of *n* instances, $\mathcal{Z} = ({\mathbf{z}}^{(c_{1})}_{t_{1}},{\mathbf{z}}^{(c_{2})}_{t_{2}}, \ldots ,{\mathbf{z}}^{(c_{n})}_{t_{n}})$, where each ${\mathbf{z}}^{(c_{i})}_{t_{i}} \equiv ({\mathbf{x}}^{(c_{i})}_{t_{i}},{\mathbf{s}}^{(c_{i})},u^{(c_{i})},t_{i})$, and the corresponding targets $\mathcal{Y} = ({\mathbf{y}}^{(c_{1})}_{t_{1}},{\mathbf{y}}^{(c_{2})}_{t_{2}}, \ldots ,{\mathbf{y}}^{(c_{n})}_{t_{n}})$. The linear coefficient matrix **B** is first estimated using a regularized least squares estimation procedure, with the *loss function* defined as:

20 $$ J({\mathbf{B}}) = \frac{1}{2n} \Vert {\mathbf{Y}} - {\mathbf{XB}} \Vert ^{2}_{F} + \alpha \lambda \Vert {\mathbf{B}} \Vert ^{2}_{F} + \alpha (1-\lambda ) \vert { \mathbf{B}} \vert , $$

where $\Vert \cdot \Vert ^{2}_{F}$ and $\vert \cdot \vert $ denote the square of the *Frobenius* norm and the $l_{1}$ norm of a matrix, respectively. **X** is the covariate matrix consisting of the covariate vectors, i.e., ${\mathbf{X}} = ({\mathbf{x}}^{(c_{1})}_{t_{1}}, {\mathbf{x}}^{(c_{2})}_{t_{2}}, \ldots , {\mathbf{x}}^{(c_{n})}_{t_{n}})^{\top}$, and **Y** is the target matrix consisting of the target vectors, i.e., ${\mathbf{Y}} = ({\mathbf{y}}^{(c_{1})}_{t_{1}}, {\mathbf{y}}^{(c_{2})}_{t_{2}}, \ldots , {\mathbf{y}}^{(c_{n})}_{t_{n}})^{\top}$. While the first term in (20) is the standard least squares loss, the second and third terms act as an *elastic-net* regularizer on the coefficients, which is employed to reduce the impact of spurious features and to avoid overfitting [47], where a model performs well for in-sample data, but does poorly for out-of-sample points. The scalars *α* and *λ* are known as the regularization parameters and are tuned using cross-validation on the training data. In this study, the tuned values for *α* and *λ* are 0.1 and 0.5, respectively. The optimization of the loss function in (20) is done using a coordinate descent algorithm.

After estimating the optimal coefficients in **B**, the hyperparameters associated with the GP are estimated by maximizing the marginal *log-likelihood* of the residuals, using the marginal likelihood in (8). For each training instance, the residual vector is defined as $\boldsymbol{\delta}^{(c_{i})}_{t_{i}} = {\mathbf{y}}^{(c_{i})}_{t_{i}} - { \mathbf{B}}^{\top}{\mathbf{x}}^{(c_{i})}_{t_{i}}$. Let $\bar{\mathcal{Z}}$ denote the training data set in which every training instance is augmented according to (15). Let $\bar{\boldsymbol{\delta}}$ be the vector containing all the scalar targets. Given that the marginalized conditional probability distribution, $(\bar{\boldsymbol{\delta}}\vert \bar{\mathcal{Z}})$ is a multivariate Gaussian with zero mean and covariance as $({\mathbf{K}}_{\bar{\mathcal{Z}},\bar{\mathcal{Z}}} + \sigma _{n}^{2}I)$ (see (8)), the marginalized log-likelihood can be expressed as:

21 $$\begin{aligned} \begin{aligned} \log{p(\bar{\boldsymbol{\delta}}\vert \bar{ \mathcal{Z}})} = {}& - \frac{1}{2}\bar{\boldsymbol{\delta}}^{\top} \bigl({\mathbf{K}}_{\bar{\mathcal{Z}}, \bar{\mathcal{Z}}}+ \sigma _{n}^{2}I \bigr)^{-1}\bar{\boldsymbol{\delta}} \\ &{} -\frac{1}{2}\log \bigl\vert \bigl({\mathbf{K}}_{\bar{\mathcal{Z}}, \bar{\mathcal{Z}}} + \sigma _{n}^{2}I\bigr) \bigr\vert - \frac{nm}{2} \log{2\pi}. \end{aligned} \end{aligned}$$

The marginalized log-likelihood is maximized with respect to the kernel hyperparameters and $\sigma _{n}$, using stochastic gradient descent [48].

#### Model inference

To infer any target for a microregion at a new time instance, we use the GP formulation to estimate the posterior distribution for the target, conditioned on the training data set, $(\mathcal{Z}, \mathcal{Y})$. Let the covariates for the test instance be denoted as ${\mathbf{z}}_{*} = ({\mathbf{x}}_{*},{\mathbf{s}}_{*},u_{*},t_{*})$. For the *o*th target, the posterior distribution of $y_{*o}$ is a Gaussian distribution, whose mean, $\bar{y}_{*o}$, and variance, $\operatorname{var}[y_{*o}]$ are given by the following expressions [40]:

22 $$\begin{aligned}& \bar{y}_{*o} = {\mathbf{b}}_{o}^{\top }{ \mathbf{x}}_{*} + {\mathbf{k}}_{*}^{\top}\bigl({ \mathbf{K}}_{\bar{\mathcal{Z}},\bar{\mathcal{Z}}} + \sigma _{n}^{2}I \bigr)^{-1} \bar{\boldsymbol{\delta}}, \end{aligned}$$

23 $$\begin{aligned}& \operatorname{var}[y_{*o}] = k_{**} - {\mathbf{k}}_{*}^{\top} \bigl({\mathbf{K}}_{ \bar{\mathcal{Z}},\bar{\mathcal{Z}}} + \sigma _{n}^{2}I \bigr)^{-1}{\mathbf{k}}_{*} + \sigma ^{2}_{n}, \end{aligned}$$

where ${\mathbf{b}}_{o}$ corresponds to the *o*th column of the coefficient matrix, **B**. The vector ${\mathbf{k}}_{*}$ contains the kernel function evaluation between every augmented training instance and the test instance, and the scalar $k_{**}$ is the kernel function evaluation for the test instance with itself.

## Results

We describe two sets of experimental results: first, *validation* results for spatial and temporal generalizability, and second, insights provided by our model. We also provide through *comparison* of our model’s performance with the class models, namely linear, Gaussian Process Regression (GPR) and Gradient Boosted Regression (GBR). While none of the previous works have used the all the datasets as described here, for the comparison, here, we use our feature set and their models. Regarding the *insights*, we provide three details: energy access estimates for 2020 for the entire country; energy access delineated by urban-rural divide and juxtaposed against the population growth

### Validation results

#### Spatial cross-validation

During each run of spatial cross-validation, the training and test sets are sampled from geographically distinct regions to mitigate the effect of spatial auto correlation and this procedure is shown to produce robust results [49]. The specific strategy for Senegal used in this study is described in [28], and ensures that during the multiple runs of CV, all microregions are represented in training and test samples.

Table 2(a) depicts the results of spatial cross-validation procedure performed for censal year (2013) and emphasizes the efficacy of our model in predicting energy access at microregions with highly significant correlations and low errors when compared to competing methods. *Spearman’s correlation* of > 0.6 indicates that rank correlations are preserved, which is important as the correct ordering of microregions is, at times, sufficient to identify the most deprived ones. The values of *Pearson’s r correlation* are much higher than rank correlation indicating the linear correspondence of the targets and model estimates. Our model predicts electricity access better than gas access. However we notice low *RMSE errors* in gas access for rural microregions than urban ones. Detailed results for all energy indicators are given in Additional file 1 Table 2.

**Table 2:** (a) Spatially cross validated (CV) results for energy access at microregions for 2013 and comparison with existing works. (b) Temporally validated nowcasts for 2020 with DHS-2019 data aggregated at regions and comparison with existing works. Corr. refers to Pearson’s r correlation, Rank corr. refers to Spearman’s rank correlation and RMSE refers to Root Mean Square Error, which is disaggregated by urban and rural areas. The target values are normalized between 0 and 1. The *p*-values for all targets is less than 0.001, unless annotated with a ‘*’. RMSE is in same units as the target, with lower values indicating a better fit. The columns highlighted Linear (EO) and Linear (NL) are comparative methods, where the former denotes a linear model with our covariates and the later denotes a linear model with only nighttime lights data. Results for past approaches on all covariates are reported in columns GPR (Gaussian Process Regression [12]) and GBR (Gradient Boosted Regression [25]). Spatial CV procedure was performed 100 times with different train-test split of the data, the standard deviation across the multiple runs is reported within simple brackets. In temporal validation, the testing data belonged to 2020 and the training data to 2013, 2015, and 2017. Since this procedure was run a single time, there are no standard deviations to be reported

	Electricity access					Clean cooking fuel access				
	*This study*	Linear (EO)	Linear (NL only)	GPR (EO)	GBR (EO)	*This study*	Linear (EO)	Linear (NL only)	GPR (EO)	GBR (EO)
(a) Spatial validation										
Corr.	**0.81 (0.11)**	0.65 (0.12)	0.67 (0.09)	0.55* (0.32)	0.79 (0.08)	**0.74 (0.23)**	0.71 (0.21)	0.74 (0.16)	0.33* (0.37)	0.72 (0.15)
Rank corr.	**0.74 (0.13)**	0.57 (0.19)	0.65 (0.17)	0.35 (0.56)	0.71 (0.11)	0.60 (0.26)	0.49 (0.21)	0.62 (0.18)	−0.05 (0.56)	**0.61 (0.20)**
RMSE (urban)	**0.22 (0.05)**	0.46 (0.21)	0.48 (0.18)	0.44 (0.21)	0.27 (0.05)	0.32 (0.13)	**0.24 (0.08)**	0.30 (0.11)	0.37 (0.18)	0.32 (0.13)
RMSE (rural)	0.20 (0.03)	0.26 (0.06)	0.19 (0.03)	0.22 (0.06)	**0.18 (0.02)**	0.08 (0.01)	0.08 (0.02)	0.08 (0.01)	**0.07 (0.01)**	**0.07 (0.01)**
(b) Temporal validation										
Corr.	**0.88**	0.72	0.48*	0.81	**0.88**	**0.92**	0.75	0.76	0.79	0.65
Rank corr.	**0.83**	0.77	0.74	0.74	0.85	**0.93**	0.81	0.84	0.80	0.77
RMSE (urban)	0.23	0.43	0.46	0.33	**0.21**	0.17	0.18	**0.16**	**0.16**	0.17
RMSE (rural)	**0.15**	0.24	0.19	0.22	0.16	0.30	0.13	**0.12**	0.15	0.31

#### Temporal validation

**Figure 1:**
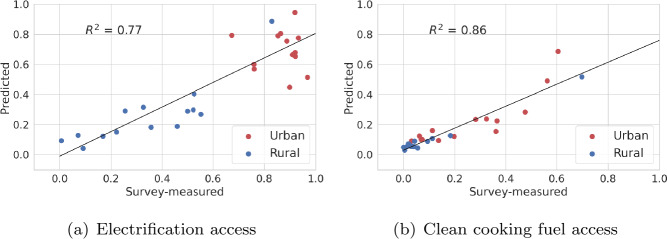
Scatter plots of temporal validation of nowcasts for 2020 with DHS derived data for 2019 for energy access at regions. Our model predictions are produced at microregions, but aggregated to regions for comparison. Targets from DHS clusters are assigned to their regions as well

We test the validity of our nowcasts by using the concurrent DHS survey. For country-wide spatial coverage, this validation is performed at regional level. The geocoded clusters from DHS are assigned to their respective regions (this mapping is already provided in DHS data). Our nowcasts are also aggregated to region level for comparison and r-squares are reported. To nowcast for 2020, our model is trained on EO data and targets for censal year (2013), as well as EO data for the years when subsequent DHS surveys are available, which are 2015 and 2017 for Senegal. Figure 1 shows that our model can explain 77% and 86% of the variation in the regional aggregates for electric and gas access, respectively.

Table 2(b) shows high *Pearson’s r and rank correlations* for both electric and gas access for temporal validation. The errors are also lower for rural areas, than urban ones. We also experiment for intercensal years, 2015 and 2017, whose estimates are validated with the DHS derived indices concurrent to those years. The details of the experimental setup and scatter plots in each case are given in Additional file 1 Table 1 and Additional file 1 Fig. 2 respectively. For both these years, we report a *r-squared* of >0.78 and >0.71 for electricity and gas access, respectively. These results state the accuracy of nowcasting abilities of our model for data-scarce situations. Our study also provides accurate nowcasts for other prominent modes of lighting and cooking, namely wood, coal and lamp, see Additional file 1 Table 3, which could help policy makers to target appropriate interventions.

Focusing on our model’s errors for 2020 for electrification access, we see that our model marginally underpredicts the electricity access for most regions irrespective of these regions being urban/rural or with high/low electricity access. The most underpredicted regions for electricity access are Kolda and Kaffrine. Doing a similar error analysis for clean cooking fuel access, we again note that our model marginally underpredicts for most regions irrespective of their urban/rural status. The urban microregions of Saint Louis, Kaolack, Thies, Louga, Fatick and rural areas of Dakar are the most underpredicted, while the urban areas of Dakar are slightly overpredicted. We would like to note that these results are data dependent, with various factors affecting the model performance with prominent ones being the noise in the EO data that is input to our model, and the quality of the surveys (temporal and spatial coverage).

### Insights into model’s intercensal estimates

#### Estimates of electricity and clean cooking fuel access for microregions in Senegal in 2020

**Figure 2:**
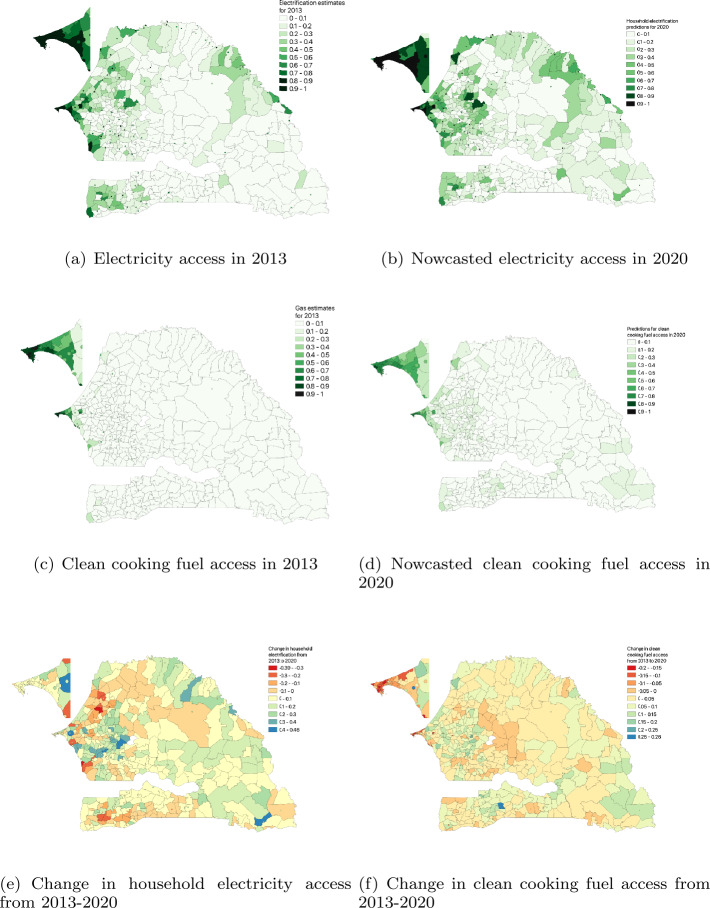
Quantifying the evolution of household energy access from 2013 to 2020 for 552 communes in Senegal. (**a**) Electricity access in 2013 using census (**b**) Nowcasted electricity access in 2020 (**c**) Clean cooking fuel access in 2013 using census (**d**) Nowcasted clean cooking fuel access in 2020 (**e**) Changes in household electricity access from 2013 to 2020. (**e**) Changes in household clean cooking fuel access from 2013 to 2020. Administratively, Senegal is organized into 14 regions and 45 departments. The finest level of policy planning is communes. From the 2013 census, it has 384 rural communes and 168 urban communes (which includes 121 urban centers). Dakar region is shown enlarged in the inset. Urban centers are shown as dots on the map

Our model’s estimates for 2020 are depicted in Fig. 2.^2^ In 2013, about 57% of households were electrified, which were mostly concentrated in the capital region of Dakar and the nearby urban area of Thies. Compared to 7% in 2013, about 11% of all rural microregions have more than half of their households electrified in 2020. The number of electrified households in urban microregions has remained the same (which amounts to about more than 85% of those microregions), even while accounting for rapid population growth in these areas.

The *change in electrification* between these years is depicted in Fig. 2(e). While, several rural areas in Kedougou and Sedhiou report positive change, it seems that electrification in some urban areas in Dakar has not kept up in 2020. We attribute it, mainly, to rapid growth of urban population in recent years, causing the electrification rate to lag or stay stagnant (further results are detailed below).

Focusing on *gas access* in Fig. 2(c), (d), (f), we notice that it was concentrated only in the urban regions of Dakar in 2013. Nationally, 67% of the households had no access to clean cooking fuels in 2013. The 2020 nowcasts show that gas access is slowly spreading to other urban centers in the country. Focusing on the urban regions, we find that about 47% of these areas have more than a quarter of their households with gas access. However, the picture looks dismal in rural microregions. Even in 2020 most of them have more than 75% of their households without access to clean cooking fuel. Figure 2(f) depicts this change and highlights the disparity between urban and rural areas, which is described in detail below.

#### Highlighting the urban-rural disparity in energy access

**Figure 3:**
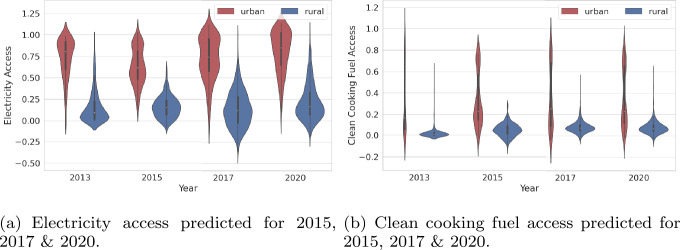
Disparity in energy access disaggregated along the urban-rural divide. Each violin plot shows the distribution of energy access for microregions for 2013, 2015, 2017 and 2020. Red denotes urban and blue denotes rural microregions

Dis-aggregated energy access along urban-rural divide from 2013-2020 is depicted in Fig. 3, where wide heterogeneity becomes evident not only between urban-rural microregions, but also within each of the urban (or rural) categories. Urban areas usually exhibit much wider energy inequities, with some of them having lower household electrification than select rural ones. However, on average, urban areas have markedly higher access to electricity than rural areas through the years. Our model reveals *stark disparities* in energy disparities even in 2020.

The spread of *gas accessibility* has a wider disparity among urban areas in 2013, with very few areas (mostly in Dakar) boasting high access to gas, while rural areas had hardly any access. It corroborates with national numbers which allocate very few households with the income to purchase clean stoves to burn gas and the recurring purchase of gas cylinders, as well as the lack of distribution outlets in far-flung rural areas [50, 51]. Our analysis reveals a very marginal increase in the gas access in rural areas in 2020.

#### Analyzing the dynamics of energy access and population growth

**Figure 4:**
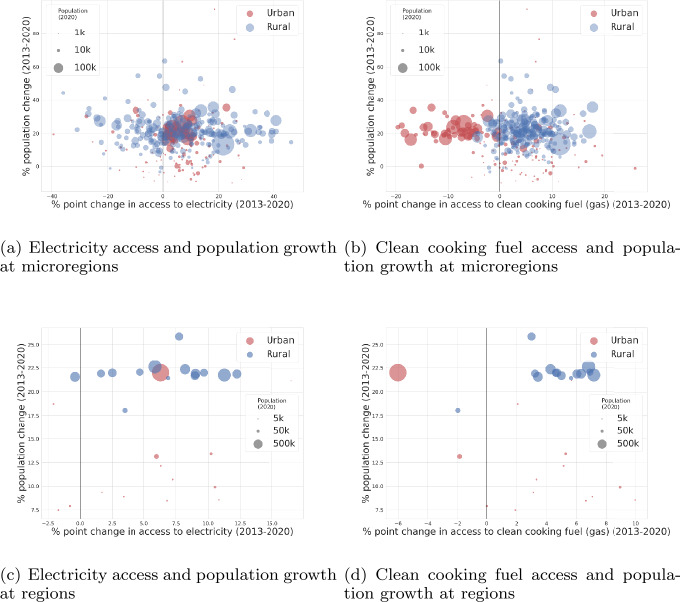
Changes in access to electricity and clean cooking fuels (in percentage point) related to population change (in percentage) from 2013-2020 at microregions (shown in (**a**) for electricity and (**b**) for clean cooking fuel) and aggregated to regions (shown in (**c**) for electricity and (**d**) for clean cooking fuel). Red points denote urban and blue for rural. Size of the circles denote their population. These plots highlight hot-spots where the rate of energy access has not kept up with its population growth

Most urban and rural areas report a positive change in electrification at regional level, see Fig. 4(c), despite their population growth, which puts Senegal in an *optimistic growth* curve. Contrasting the regional plots with micro-regional ones elucidates the point that several heterogenieties are lost when data is aggregated to sub-national levels. For microregions, we notice that urban areas have a broader horizontal spread, in both electricity and gas access, highlighting the existence of disparities within these areas, even with similar percentage population growth.

Figure 4(b) depicts that urban microregions show a negative percentage point change in gas access, highlighting that it has not kept up with population growth in urban areas. Most rural areas show a positive percentage point change in energy access highlighting that gas access is beginning to pick up in these areas even with the increased population growth. Note that most of the rural areas had no access to gas as cooking fuel in 2013, and thus they exhibit marked percentage point change in Figs. 4(c) and (d).

## Discussion, limitations and conclusions

The objective of our EO-data based modeling approach is to provide microestimates when surveys are unavailable, e.g. during intercensal periods or in regions of conflict or those recovering from natural disasters or political upheaval and, thus, to augment the existing surveying efforts on the ground.

**Figure 5:**
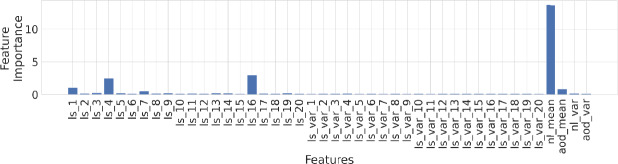
Feature relevance obtained using *Automatic Relevance Determination* (ARD) kernel, which is often used for this task in GPs, where the inverse length scale parameter of each input variable is used as a proxy for feature relevance [40]. *nl* refers to nighttime lights, *aod* refers to aerosol optical depth and *ls* refers to landsat features

The basic premise of using heterogeneous satellite data is the assumption that they can capture the heterogenieties in energy access on the ground, possibly via nighttime luminosity data or urban-buildup. To identify which input features are most useful, we perform *feature selection* using ARD kernel, and the top features deemed important are, indeed, nighttime lights, selected Landsat-8 features and aerosol data, as shown in Fig. 5.

**Figure 6:**
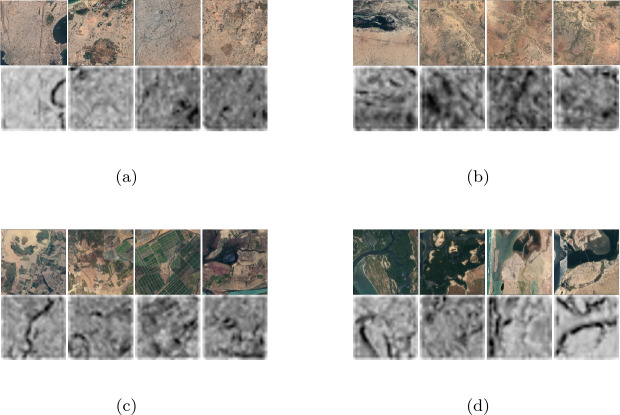
Visualizing the maximally activating Landsat images for four selected features extracted by the deep neural network. Each pair in each sub-figure shows the original Landsat image (*top*) and the corresponding activation map for a given feature (*bottom*). The four selected features appear to correspond to presence of (**a**) dense urban areas, (**b**) sparse rural areas, (**c**) agricultural land, and, (**d**) water bodies. See Additional file 1 Section “Visualization of Deep Neural Network Features” for more details

*Visualizations* of the features extracted using Landsat-8 imagery point to semantically meaningful ones, likely capturing urban areas, sparse rural settlements, agricultural and presence of water, shown in Fig. 6. Though these selected features are specific to the EO data and country analyzed here, they conform to the broader consensus of existing research with nightlights as the most important feature [10, 35–37].

Our model *leverages* EO and DHS data for all past years when available. This involves allocating yearly DHS clusters to nearest microregions. Our allocation mechanism is *robust* to the inherent noise in spatial locations of DHS clusters, whose geo-coordinates are moved to protect privacy (clusters in urban areas are moved by up to 2 km and those in rural by 10 km). Contrasting, most existing works rely on extracting satellite imagery (which is usually at 30 sq. m resolution) around the DHS clusters and are, thus, susceptible to learning from noisy or misaligned data [7, 14, 31].

Validation at intercensal microestimates and nowcasts remain a challenge, given the lack of fine-grained ground truth data. We mitigated it by providing validation of our nowcasts at regional level using temporally closest DHS data. The next census of Senegal (likely scheduled for 2023) or more local data will likely provide a good validation point.

Regarding the generalizability of our model to other countries, we do believe that our methodology can be replicated to our countries, given the availability of their EO data and targets for training purposes (so that the model will learn country-specific relationships). We understand that significant gaps in both temporal and spatial coverage of surveys do exist for many countries, however our methodology is not dependent on availability of surveys with uniform temporal regularity and spatial coverage. Our model can be trained with the existing survey data and EO data for a country to nowcast. The kernel function, that lies at the core of our modeling approach, is designed to appropriately weight the temporal and spatial information in the surveys (i.e., more weight to more recent survey data).

Another research avenue that is worth exploring regarding generalizability is how well does our model that is trained on one country, perform for another country, especially neighboring countries.

### Limitations

There are limitations to employing nighttime light data to accurately measure aspects of human development, including access to electrification, which was predicted less successfully for some countries than others, as shown in [52]. Researchers have highlighted the limitations of existing models that learn solely from nighttime imagery, particularly their tendency to generally under-perform in differentiating deprived (or poor) from the critically deprived (or ultra-poor) regions, as shown in the context of Sub-Saharan Africa [53]. Researchers have also demonstrated the susceptibility of such models to inherent noise in the data [54].

While our model leverages additional input data besides nighttime lights, more concerted research efforts are needed to comprehensively understand its performance and generalizability. Satellite imagery, especially at the resolution analyzed in this work, might not be able to distinguish between subtle nuances of urban and peri-urban areas (e.g. the presence of slums or unauthorized settlements), as highlighted by [7] and, thus, would be weak in distinguishing energy access in such microregions.

Recent works also highlight important concerns related to the presence of bias when human developmental indicators, notably poverty and electrification are predicted using nighttime lights [55–57]. We are currently working to understand the fairness aspects of our model, so that our microestimates can be trusted and used by policy-makers.

### Future directions

While this paper focuses on Senegal, the proposed framework can also be developed for other countries, by training the model using the EO data and energy targets for that country, so that it can learn country-specific mappings and produce the desired microestimates. With many geo-located household surveys being conducted regularly and cheap availability of EO data, our framework has the potential to provide a cheap and good approximation, and continuous monitoring for intercensal statistics at the microregional level and can supplement the surveying tasks for better tracking of SDG 7.

## Supplementary Information

Below is the link to the electronic supplementary material.

Supplementary information. Provides additional information about extracting targets from census data and additional results (PDF 1.6 MB)

## Data Availability

The census data can be obtained by contacting Dr. Emmanuel Letouze (eletouze@datapopalliance.org). The DHS data used, in this work, can be obtained by registering at https://dhsprogram.com/. The links to downloading EO data are given in Methods. The associated code to generate the results in this manuscript is available for review at https://github.com/neetip/energy_access.

## Footnotes

1 Last census for Senegal was done in 2013

2 Here, clean cooking fuel refers to use of liquefied petroleum gas or electricity used for cooking at household level, and is, henceforth, referred as gas in our paper.
