# Supplementary material for: Accurate intercensal estimates of energy access to track Sustainable Development Goal 7
Source: EPJ Data Sci. 2022 Dec 9;11(1):60. doi: 10.1140/epjds/s13688-022-00371-5 (PMC9734985; doi:10.1140/epjds/s13688-022-00371-5)
Supplement: Supplementary file 1 — Supplementary information. Provides additional information about extracting targets from census data and additional results (PDF 1.6 MB) [file 13688_2022_371_MOESM1_ESM.pdf]

SUPPLEMENTARY INFORMATION

Accurate Intercensal Estimates of Energy Access to Track Sustainable Development Goal 7

Neeti Pokhriyal[1*], Emmanuel Letouzé[2,3] and Soroush Vosoughi[1]

*Correspondence:
neeti.pokhriyal@dartmouth.edu
[1] Dartmouth College, Hanover, USA
Full list of author information is available at the end of the article

Mapping of census responses

The mapping of each of the census response variable to accessibility vector is as follows: For lighting, the census response variable 'Eléctricité' is mapped to our regression target 'Electric'; 'Lampe rechargeable' is mapped to 'Lamp'; 'Lampe tempête', 'Bougie', 'Bois', 'Lampe à pétrole artisanale' to 'Candle'; all other responses are mapped to 'Other lighting'. For cooking fuel,'Gaz' and 'Eléctricité' to 'Gas'; 'Charbon' to 'Coal'; 'Bois' to 'Wood', and all other responses to 'Other cooking fuels'. We note that there is a 0.76 positive correlation between households that use electricity for lighting and gas for cooking, and high negative correlation of -0.93 between households that use gas and households that use wood as cooking fuel. Our model exploits these cross-correlations among the different lighting and cooking fuel variables, which are modeled as multiple targets in our regression equation and their relationship to EO data is learnt jointly to provide accurate estimates. The cross-correlations among the targets are shown in Supplementary Figure 1 (b). It is important to note that target values lie between 0 and 1 and each is spread differently throughout the country in 2013, as depicted in Supplementary Figure 1 (c).

Validation of model's estimates for various inter-censal years

Table 1 details the experimental setup to test the robustness of our model's intercensal estimates, since the last census for Senegal in 2013 till 2020. Figure 2 shows the scatter plots of how our model performs compared to DHS derived indices. We also notice that the uncertainties along with our model estimates are lower for gas access than electricity access for all the intercensal years, signifying more confidence of our model in making those predictions.

	Training	Testing	Validation	R-squared for electricity	R-squared for gas
1	2013	2015	2015	0.78	0.72
2	2013 + 2015	2017	2017	0.80	0.71
3	2013 + 2015 + 2017	2020	2019	0.77	0.86

Table 1 Experimental setup for checking the robustness of our model's intercensal estimates. The "Training" columns denotes the years corresponding to training data, which is done using EO data and either census (for 2013) or DHS targets (which are extracted for 2015, 2017 and 2019). The "Testing" column details the years for which model estimates were generated, while the "Validation" column denotes the years for which these estimates are validated. The "R-squared" column details how our model performs for different inter-censal years.

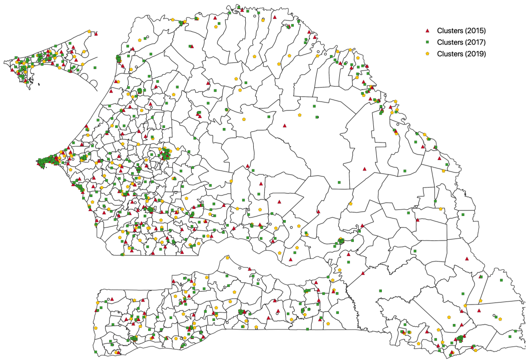

(a) Locations for DHS clusters for 2015, 2017 and 2019 superimposed on the Senegal micro-regional (commune) boundaries

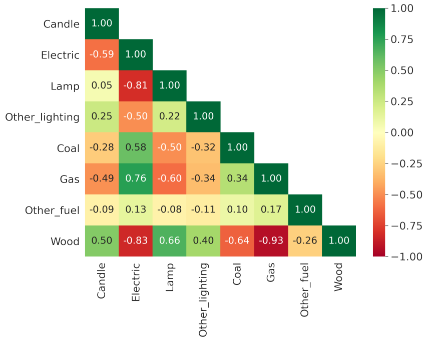

(b) Correlations among the targets from 2013 census

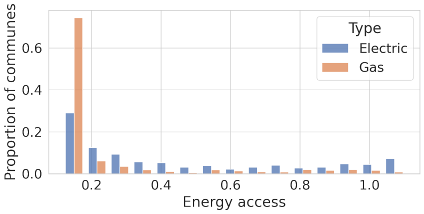

(c) Distribution of the spread of electricity for lighting and gas for cooking from 2013 census.

Figure 1 Multi-year DHS cluster locations and details about the targets from 2013 census for Senegal

	Lighting Source				Cooking Fuel			
	Candle*	Electric	Lamp	Other lighting*	Coal*	Gas	Wood	Other fuel*
Corr.<br>p-value	0.51 (0.18)<br>0.03	0.81 (0.11)<br>< 0.001	0.60 (0.10)<br>< 0.001	0.21 (0.24)<br>0.04	0.41 (0.12)<br>0.02	0.74 (0.23)<br>< 0.001	0.85 (0.10)<br>< 0.001	0.09 (0.16)<br>0.21
Rank corr.<br>p-value	0.53 (0.19)<br>0.02	0.74 (0.13)<br>< 0.001	0.61 (0.10)<br>< 0.001	0.22 (0.30)<br>0.07	0.48 (0.21)<br>0.04	0.60 (0.26)<br>< 0.01	0.69 (0.17)<br>< 0.001	0.16 (0.20)<br>0.11
RMSE (all)	0.15 (0.03)	0.21 (0.03)	0.20 (0.03)	0.08 (0.02)	0.12 (0.01)	0.21 (0.08)	0.20 (0.06)	0.05 (0.02)
RMSE (urban)	0.10 (0.02)	0.22 (0.05)	0.16 (0.03)	0.06 (0.02)	0.17 (0.02)	0.32 (0.13)	0.29 (0.11)	0.05 (0.02)
RMSE (rural)	0.17 (0.03)	0.20 (0.03)	0.23 (0.04)	0.08 (0.01)	0.07 (0.01)	0.08 (0.01)	0.11 (0.02)	0.05 (0.01)

Table 2 Spatially cross validated results for energy access at microregions in Senegal for 2013. Corr refers to Pearson's r correlation, Rank corr refers to Spearman's rank correlation and RMSE refers to Root Mean Square Error, which is disaggregated by urban and rural areas. The target values are normalized between 0 and 1. The p-values for all targets less than < 0.001. The standard deviation associated with the multiple runs for each measurement is reported within simple brackets.

	Lighting Source				Cooking Fuel			
	Candle*	Electric	Lamp	Other_lighting	Coal	Gas	Wood	Other_fuel*
Corr.<br>p-value	0.33<br>0.09	0.88<br>< 0.001	0.83<br>< 0.001	0.77<br>< 0.001	0.64<br>< 0.001	0.92<br>< 0.001	0.88<br>< 0.001	0.18<br>0.37
Rank corr.<br>p-value	0.46<br>0.01	0.83<br>< 0.001	0.81<br>< 0.001	0.68<br>< 0.001	0.70<br>< 0.001	0.93<br>< 0.001	0.94<br>< 0.001	0.33<br>0.09
RMSE (all)	0.10	0.19	0.20	0.09	0.17	0.08	0.21	0.05
RMSE (urban)	0.11	0.23	0.21	0.04	0.22	0.10	0.28	0.07
RMSE (rural)	0.08	0.15	0.20	0.12	0.08	0.06	0.11	0.03

Table 3 Temporal validation of nowcasts for 2020 with DHS derived estimates of energy access at regional level. Corr. refers to Pearson's r correlation, Rank corr. refers to Spearman's rank correlation and RMSE refers to Root Mean Square Error, which is disaggregated by urban and rural areas. The target values are normalized between 0 and 1.

Visualization of Deep Neural Network Features

The ResNet-18 neural network used in this work transforms each Landsat-8 satellite image into a 512 length feature vector. One way to understand the semantic significance of each feature is to understand what parts of the original image contribute to the calculation of each feature, also referred to as an activation map [1, 2]. For each feature, we find the images that activate the feature most strongly. These images and the corresponding activation maps allow us to semantically interpret the feature. In Figure 5 of the main paper, we show the maximally activating images and their corresponding activation maps for four features that appear to correspond to presence of dense urban areas, sparse rural areas, agricultural land, and water bodies.

Author details
[1] Dartmouth College, Hanover, USA. [2]Data-Pop Alliance, New York, USA. [3]University Pompeu Fabra, Barcelona, Spain.

References
1. Jean, N., Burke, M., Xie, M., Davis, W.M., Lobell, D.B., Ermon, S.: Combining satellite imagery and machine learning to predict poverty. Science **353**(6301), 790–794 (2016)
2. Xie, M., Jean, N., Burke, M., Lobell, D., Ermon, S.: Transfer learning from deep features for remote sensing and poverty mapping. In: 30th AAAI Conference on Artificial Intelligence (2016)

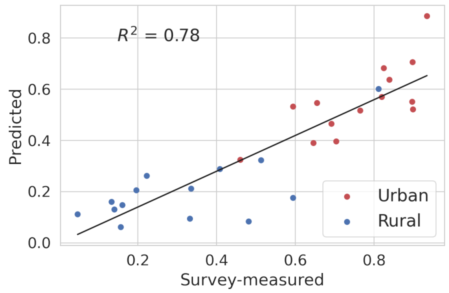

(a) Scatter plot for electricity access in 2015

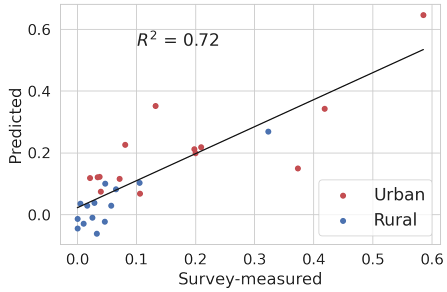

(b) Scatter plot for gas access in 2015

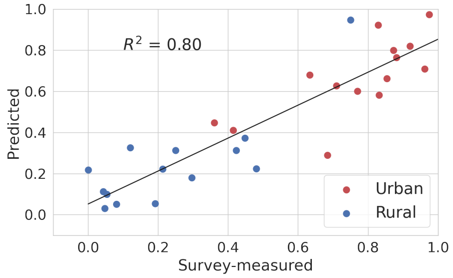

(c) Scatter plot for electricity access in 2017

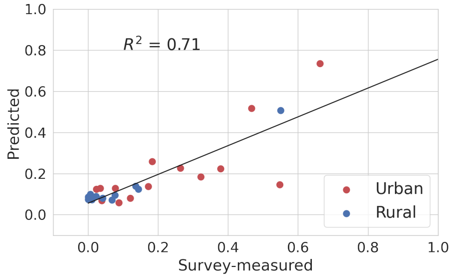

(d) Scatter plot for gas access in 2017

Figure 2 Scatter plots of model estimates (given on y-axis) with survey (DHS) derived indices (given on x-axis) for inter-censal years 2015 and 2017. R-squared values are calculated for each year, with rural regions denoted as blue and urban ones as red. Over the years, our model does better in estimating the electricity access across the population than clean cooking fuel (gas) access.
